# Supplementary material for: Synthesis of Carvacrol Derivatives as Potential New Anticancer Agent against Lung Cancer
Source: Molecules. 2022 Jul 19;27(14):4597. doi: 10.3390/molecules27144597 (PMC9323284; doi:10.3390/molecules27144597)
Supplement: Supplementary file 1 [file molecules-27-04597-s001.zip › molecules-1813309-supplementary.pdf]

molecules

MDPI

Supplementary Materials

Article

Synthesis of Carvacrol Derivatives as Potential New Anticancer Agent Against Lung Cancer

Anu Bansal [1], Md. Moshfekus Saleh-E-In [2], Pallab Kar [3], Ayan Roy[4] and Neeta Raj Sharma [1,*]

1. School of Bioengineering and Biosciences, Lovely Professional University, Phagwara, 144411, India; anu.21971@lpu.co.in (A.B.); neeta.raj@lpu.co.in (N.R.S.)
2. Division of Forest Resources, College of Forest and Environmental Sciences, Kangwon National University, Chunchon-200701, Republic of Korea; saleheinm saleheinm@kangwon.ac.kr (M.M.S.-E.-I.)
3. B.S. Diagnostic and Pathology Laboratory, Siliguri-734001, India; pallabkar.bio@gmail.com (P.K.)
4. Mailman School of Public Health, Columbia University, New York 10032, USA; ayanroy.bio@gmail.com (A.R.)

*Correspondence: neeta.raj@lpu.co.in (N.R.S.); Tel.: +918283921144

3. Materials and Methods

3.1. Chemicals and reagents

In the present study, all of the reagents and chemicals were procured from Sigma-Aldrich of AR grade.

3.2. Synthesis of carvacrol aldehyde (2-hydroxy-3-methyl-6-(propan-2-yl)benzaldehyde)

Periodically, 8.1 g of triethylamine (Et_3N), 4.8g of anhydrous stannous chloride ($SnCl_2$) were mixed with 210 mL of toluene. The reaction mixture was stirred continuously for 20 min followed by the addition of 30 g carvacrol under reflux at 50-60 °C. Thereafter, the intermediate (organic layer) was producedand mixed with paraformaldehyde (13.8 g) followed by refluxing at 50-60 °C for 2-3 hcontinuously [1].

3.3. Synthesis of carvacrol derived Schiff base

Carvacrol aldehyde (100 mg) and 2-aminophenol (60 mg) were dissolved in 5 mL of ethanol and stirred for 15 min followed by dropwise addition of concentrated HCl. Gradually, the temperature was increased to 80 °C. The aqueous layer was separated and washed with 10 mL of demineralized water. Subsequently, ethyl acetate (10 mL) was added and washed with brine solution (10 mL). The compound was filtered through sodium sulphate (Na_2SO_4) (10 gm) and precipitate was vacuumed below the temperature 50 °C. Finally, pure orange color Schiff base (yield: 120 mg) was recovered and collected [2].

3.4. Synthesis of Cu (II)-Schiff base complex

Schiff base ligand (200 mg) and copper acetate (50 mg) were added in ethanol solution (10 mL) and continuously stirredat 25-30 °C for 10-15 min. The mixture was further heated to 80-85 °C for the next 6 h. The mixture was gradually cooled down at room temperature (25 °C) which produced a precipitate product with green color. Then the precipitate was filtered and washed withpre-cooled ethanol for further analysis [3].

2. Results and Discussion

2.1. Synthesis of carvacrol aldehyde (2-hydroxy-3-methyl-6-(propan-2-yl) benzaldehyde)

In the synthesis process, acetylation and alkylation took place in the presence of $SnCl_2$, Et_3N and paraformaldehyde [$(CH_2O)n$] to maximize the yield of aldehyde product from the reaction exclusively at the ortho-position [1]. The ortho chelation in carvacrol is provided by $SnCl_2$ due to the charge density, further stabilized by Et_3N. The paraformaldehyde acts as an electrophile agent in this reaction [4].

Carvacrol acts as a weak acid and lost its hydroxyl proton during formylation which leads to the generation of carvacrol-anion. Thereafter, the electron density is increased on the benzene ring of carvacrol. Reaction with $SnCl_2$ leads to the formation of carvacrol-$SnCl_2$ complex as an intermediate alcohol derivative. Further, reaction with paraformaldehyde causes the oxidation of alcoholic group on carvacrol-$SnCl_2$ complex followed by the conversion of paraformaldehyde to a methyl derivative during the reduction process. Aldehyde group of carvacrol-$SnCl_2$ complex is donated by paraformaldehyde specifically at ortho position of the carvacrol. As a result, a paleyellow liquid of carvacrol aldehyde was obtained. The product of carvacrol aldehyde synthesis scheme has been provided (Scheme 1).

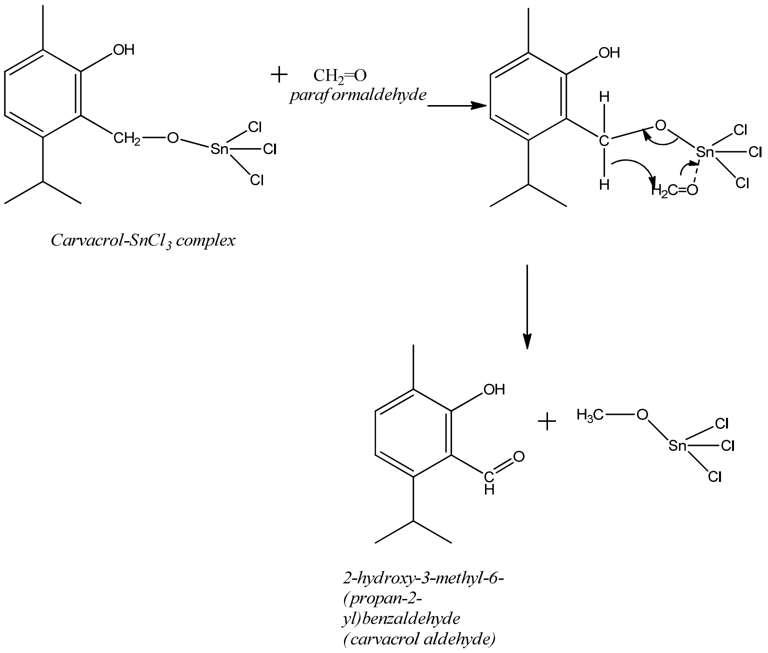

Scheme S1: *ortho*-Formylation with paraformaldehyde of carvacrol

The characterization of carvacrol aldehyde was done with the help of ^{1}H-NMR, mass spectra and FTIR. To compare the structure of the pure compound with the reference ^{1}H-NMR spectrum, it showed the presence of an aromatic compound indicated by a sharp peakat 7.9 ppm and showedthe substitution of the hydroxyl group in the aromaticring. The sharp peaks at 1.5-2.2 ppm indicated the presence of a methyl group in the structure. The peak at 10.3 ppm notified the presence of aldehyde group whereas the hydroxyl group (OH) was observed at 13.17 ppm. The molecular weight of the structure was confirmed by the ion peak at m/z 179.30 by mass spectrum. The FT-IR spectra showed significant peaks with stretching band which correspond to the aromatic C-H bond at ν 2965 cm^{-1}, whereas the bands at ν 2926 cm^{-1} correspond to the CH group of aldehyde. The carbonyl stretching frequencies were observed at ν 1627 cm^{-1}. The spectroscopic data of carvacrol aldehydehas been provided in the Supplementary Materials (Figures S1-S3).

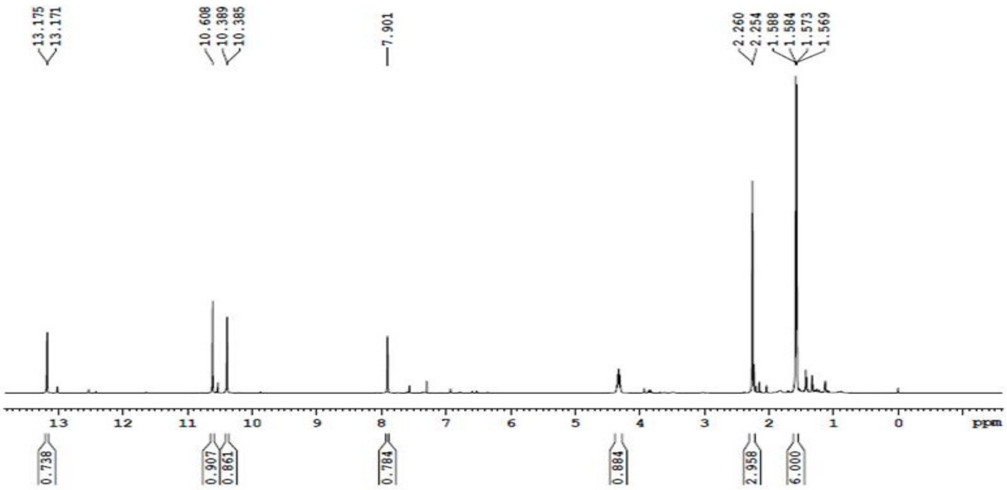

Figure S1: ^{1}H-NMR spectrum of 2-hydroxy-3-methyl-6-(propan-2-yl) benzaldehyde (carvacrol aldehyde).

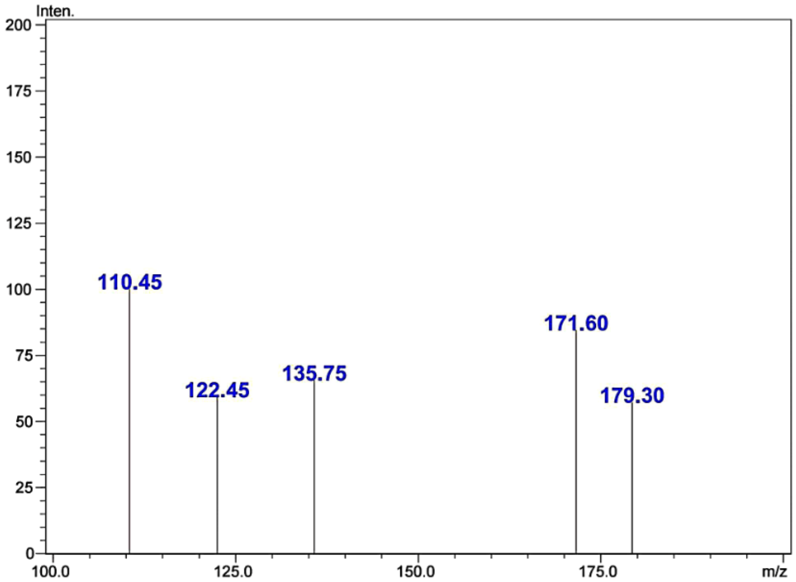

Figure S2: Mass Spectra of 2-hydroxy-3-methyl-6-(propan-2-yl) benzaldehyde (carvacrol aldehyde).

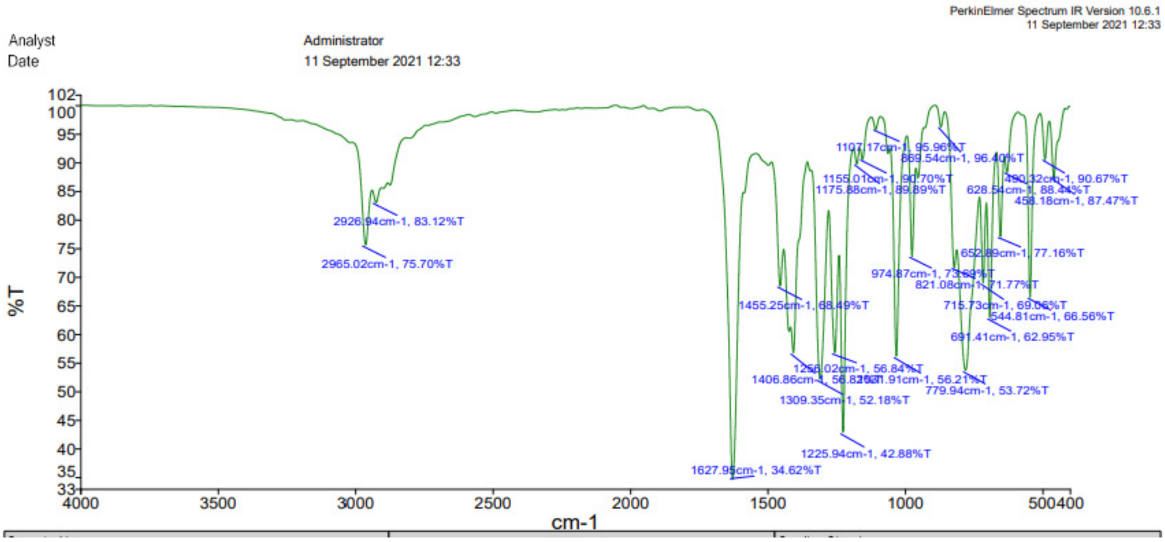

Figure S3: FT-IR Spectrum of 2-hydroxy-3-methyl-6-(propan-2-yl) benzaldehyde.

2.2. Synthesis of carvacrol derived Schiff base

The existing studies are emphasizing more towards the development of Schiff bases derived from the aromatic aldehyde ortho substituted with a hydroxyl (-OH) group because they have the ability to serve as a bidentate ligand for transition metal ions [5]. In the present study, we synthesized the Schiff base from a mixture of carvacrol aldehyde and 2-aminophenol in ethanol followed by the addition of concentrated HCl at 75-80 °C. Following the various steps, the Schiff base (yield: 120 mg) was recovered. The molecular weight was confirmed by the mass spectrum with a molecular ion peak at m/z 269. The FT-IR spectra of the Schiff base showed significant peaks at ν 2959 cm-1which was the corresponding aromatic C-H bonds with stretching vibration. Moreover, the other bands corresponding to aldehydic C=N, C=C and N-O groups werealso observed at 1593 cm^{-1}, 1556 cm^{-1} and 1223 cm^{-1} respectively. The spectroscopic data of Carvacrol derived Schiff base has been provided in the Supplementary Materials (Figure S4 and S5). The scheme (Scheme 2) of carvacrol derived schiff base synthesis has been provided.

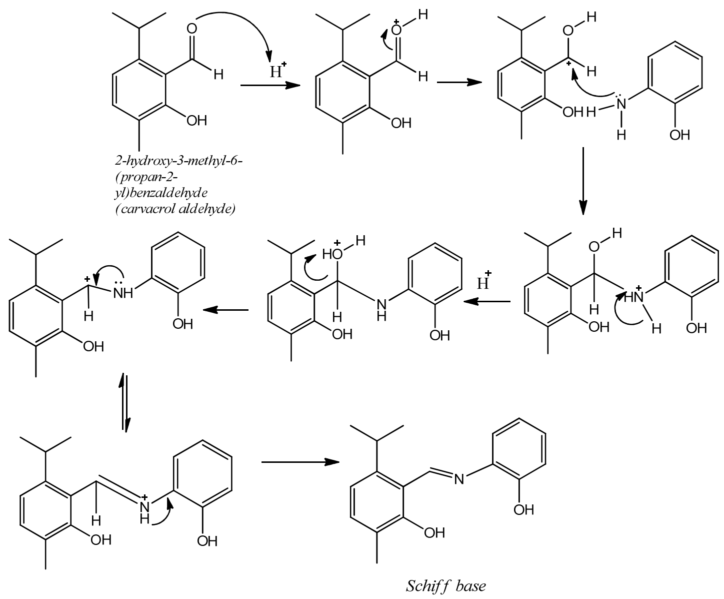

Scheme S2: Synthesis of Schiff base using carvacrol aldehyde

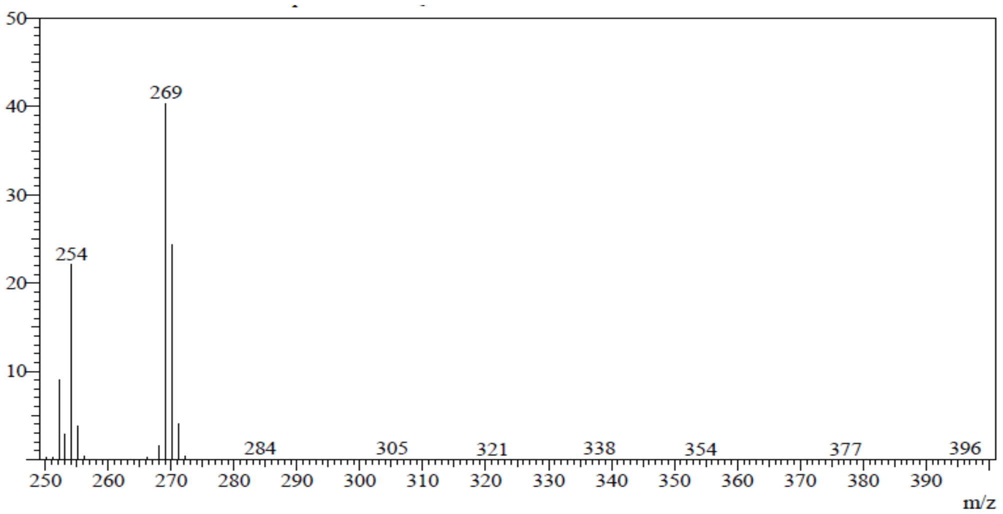

Figure S4: Mass Spectra of Schiff base.

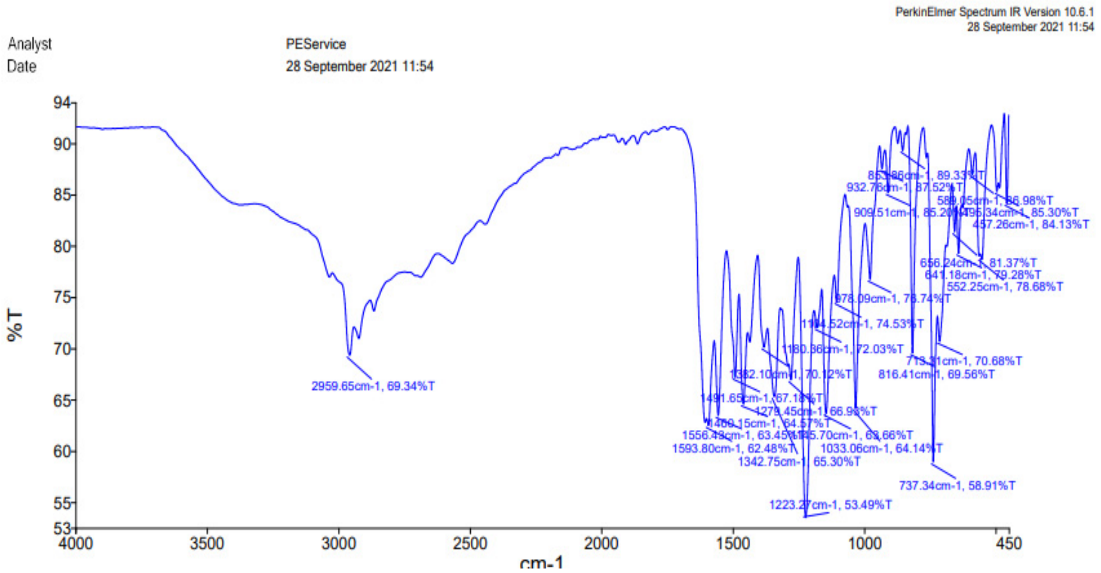

Figure S5: FT-IR spectrum of Schiff base.

2.3. Synthesis of Cu (II)-Schiff base complex

In the present study, green color crystals of Copper Schiff base complex (100 mg) was synthesized using the Schiff base ligand derived from carvacrol (Figure 2). The surface morphology of the complex of Copper Schiff base was studies through SEM and EDX (Figures S6a and S6b) The SEM micrographs revealed that the complex possesses uniform surface. The EDX peaks confirmed the occurrence of copper element in the complex. The elemental composition along with its relative proportion shown by EDX analysis. The SEM and EDX analysis confirmed the copper percentage (4.99%) and weight percentage (21.03%) respectively. The sharp peaks of XRD patterns indicated the crystalline nature of the Copper Schiff base complex that recorded at the range of 2θ = 2–60° (Figure S7). The Copper Schiff base complex displayed the peak at 2θ = 7°, 10°, 11°, and 18°. Similarly, Cu(II) complex with bidentate N_2O_2 Schiff base ligand showed the peaks at 2θ = 10°, 11°, 17°, 19°, and 21°. This might be attributed that our findings are in support of earlier studies and confirmed the formation of Copper Schiff based complex [6].

Zeta potential is used to measure surface charges on the compounds and determine their degree of repulsion between one another in a suspension. By virtue of the electrostatic repulsion among individual particles in suspension, a good stability was indicatedby the zeta potential as <-30 mV or > +30 mV,of particles in suspension solution. To determine the stability of Copper Schiff base complex, it was dispersed in ethanol at 30°C and analyzed (Figure S8). In the result, three peaks were observed, peak one had an average of 34.3 ± 49.3 mV, and its area contributed to 53% of the potential distributions. The second peak was at -94.4 ± 35.2 mV with a contributed area of 33.8% of the potential distributions. The third peak observed at 122 ± 12.9 mV, and its area contributed to 13.2% of the potential distributions. The average of all the analyzed peaks was -53.5 mV. These results indicated that Copper Schiff base complex was stable.

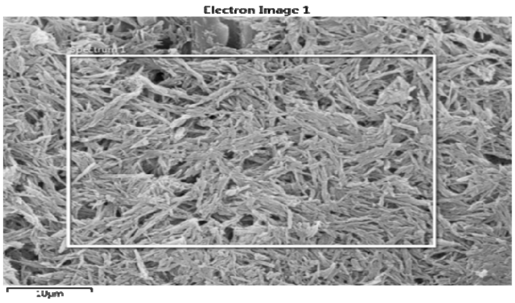

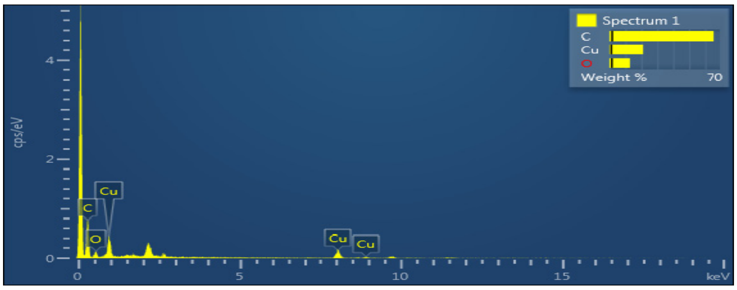

(A) **(B)**

Figure S6: (a) Scanning electron microscopy (SEM) and **(b)** SEM-energy-dispersive X-Ray (EDX) analysis of Copper Schiff base complex.

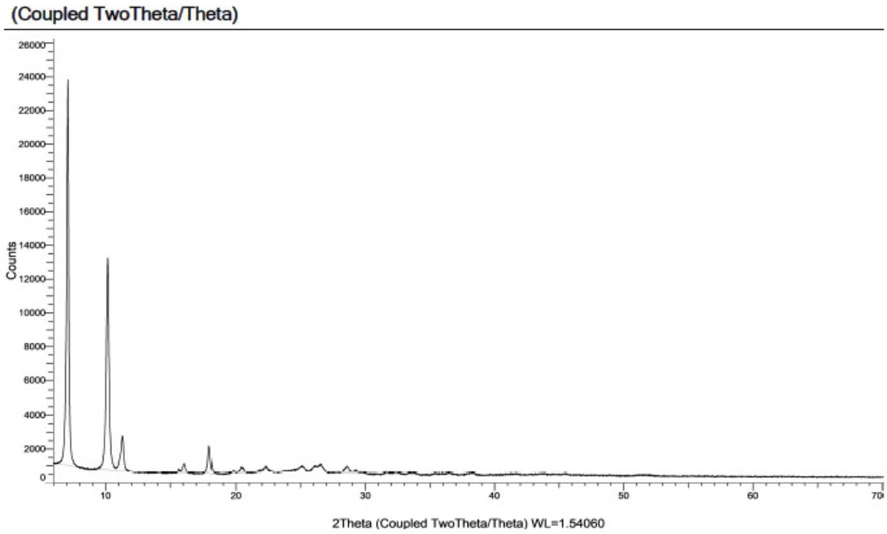

Figure S7: Powdered XRD Spectra of Copper Schiff base complex.

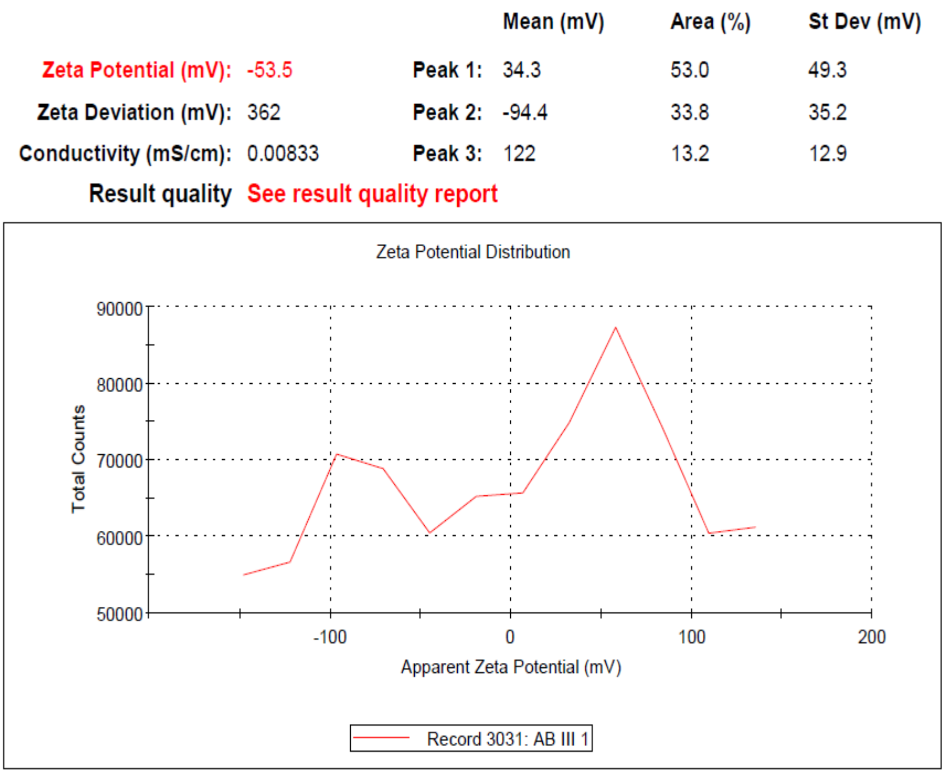

Figure S8: Zeta potential analysis of copper Schiff base complex.

Table S1: Reagents used for PCR.

Sn.	Components	Amount (50 µl) for single PCR reaction	Final concentration in the PCR reaction
**1.**	Water	30 µl	-
**2.**	10X PCR buffer	5µl	1X
**3.**	dNTP solution (2 mM)	5µl	0.2 mM of each dNTP
**4.**	25 mM $MgCl_2$ solution	3µl	1.5 mM
**5.**	Bax, Bcl-2, caspase-3 and caspase-9 (sense and antisense) PCR Primers set, 10 pmole/µ	2µl + 2µl	0.4 µM
**6.**	cDNA*	2µl	~30 ng
**7.**	Taq DNA Polymerase, 5 units/µl	1µl	0.1 units/µl
	**Total volume**	**50 µl**	NA

Table S2: Primers for reverse transcription-PCR (RT-PCR).

Gene	Primer Sequence (5'-3')	Amplification Condition
**Bax**	CAT CTT CTT CCA GAT GGT GA<br>GTT TCA TCC AGG ATC GAG CAG	95° C for 2 min<br>94° C for 45 sec<br>53° C for 45 sec x 30cycles<br>72° C for 1.5 min<br>72° C for 7 min
**bcl-2**	GAG ACA GCC AGG AGA AAT CA<br>CCT GTG GAT GAC TGA GTA CC	95° C for 2 min<br>94° C for 45 sec<br>53° C for 45 sec x 30cycles<br>72° C for 1.5 min<br>72° C for 7 min
**Caspase 3**	CCTCAGAGAGACATTCATGG<br>GCAGTAGTCGCCTCTGAAGA	95° C for 2 min<br>94° C for 45 sec<br>53° C for 45 sec x 30cycles<br>72° C for 1.5 min<br>72° C for 7 min
**Caspase 9**	AGTTCCCGGGTGTCGTCTAT<br>GCCATGGTCTTTCTGCTCAC	95° C for 2 min<br>94° C for 45 sec<br>53° C for 45 sec x 30cycles<br>72° C for 1.5 min<br>72° C for 7 min

References:

1. Hansen Vidar, T.; Skattebøl, L. Ortho-formylation of phenols; preparation of 3-bromosalicylaldehyde. *Org. Synth.* **2005**, 82, 64.
2. Hassan, A.M.; Nassar, A.M.; Hussien, Y.Z.; Elkmash, A.N. Synthesis, Characterization and Biological Evaluation of Fe (III), Co (II), Ni(II), Cu(II), and Zn(II) Complexes with Tetradentate Schiff Base Ligand Derived from Protocatechualdehyde with 2- Aminophenol. *Appl. Biochem. Biotechnol.* **2012**, *167*, 581–594.
3. Taş, E.; Kasumov, V.T.; Şahin, Ö.; Özdemir, M. Transition metal complexes with tridentate salicylaldimine derived from 3, 5-di- t-butylsalicylaldehyde. *Transit. Met. Chem.* **2002**, *27(4)*, pp.442-446.
4. Hansen, T.V.; Skattebøl, L. Discussion addendum for: ortho-formylations of phenols; preparation of 3-Bromosalicylaldehyde. *Org. Synth.* **2012**, *89*, 220-229.
5. Roman, G.; Romani', G.; Andree, M. New Schiff Bases from Ortho-Hydroxyaryl Aldehydes Thiophene Derivatives View Project Design of Isoselective Heme Oxygenase Inhibitors View Project New Schiff Bases from Ortho-Hydroxy Aryl Aldehydes. *Artic. Maced. J. Chem. Chem. Eng.* **2001**, *20*, 131–136.
6. Guo, Y.; Hu, X.; Zhang, X.; Pu, X.; Wang, Y. The Synthesis of a Cu(II) Schiff Base Complex Using a Bidentate N2O2 Donor Ligand: Crystal Structure, Photophysical Properties, and Antibacterial Activities. *RSC Adv.* **2019**, *9*, 41737–41744.
